# Cinacalcet use in secondary hyperparathyroidism: a machine learning-based systematic review

**DOI:** 10.3389/fendo.2023.1146955

**Published:** 2023-07-19

**Authors:** Xiaosong Li, Wei Ding, Hong Zhang

**Affiliations:** Department of Thyroid Surgery, The Second hospital of Jilin University, Changchun, Jilin, China

**Keywords:** calcimimetics, FGF-23, bibliometrics, LDA analysis, machine learning

## Abstract

**Introduction:**

This study aimed to systematically review research on cinacalcet and secondary hyperparathyroidism (SHPT) using machine learning-based statistical analyses.

**Methods:**

Publications indexed in the Web of Science Core Collection database on Cinacalcet and SHPT published between 2000 and 2022 were retrieved. The R package “Bibliometrix,” VOSviewer, CiteSpace, meta, and latent Dirichlet allocation (LDA) in Python were used to generate bibliometric and meta-analytical results.

**Results:**

A total of 959 articles were included in our bibliometric analysis. In total, 3753 scholars from 54 countries contributed to this field of research. The United States, Japan, and China were found to be among the three most productive countries worldwide. Three Japanese institutions (Showa University, Tokai University, and Kobe University) published the most articles on Cinacalcet and SHPT. Fukagawa, M.; Chertow, G.M.; Goodman W.G. were the three authors who published the most articles in this field. Most articles were published in *Nephrology Dialysis Transplantation*, *Kidney International*, and *Therapeutic Apheresis and Dialysis*. Research on Cinacalcet and SHPT has mainly included three topics: 1) comparative effects of various treatments, 2) the safety and efficacy of cinacalcet, and 3) fibroblast growth factor-23 (FGF-23). Integrated treatments, cinacalcet use in pediatric chronic kidney disease, and new therapeutic targets are emerging research hotspots. Through a meta-analysis, we confirmed the effects of Cinacalcet on reducing serum PTH (*SMD* = -0.56, 95% *CI* = -0.76 to -0.37, *p* = 0.001) and calcium (*SMD* = -0.93, 95% *CI* = -1.21to -0.64, *p* = 0.001) and improving phosphate (*SMD* = 0.17, 95% *CI* = -0.33 to -0.01, *p* = 0.033) and calcium-phosphate product levels (*SMD* = -0.49, 95% *CI* = -0.71 to -0.28, *p* = 0.001); we found no difference in all-cause mortality (*RR* = 0.97, 95% *CI* = 0.90 to 1.05, *p* = 0.47), cardiovascular mortality (*RR* = 0.69, 95% *CI* = 0.36 to 1.31, *p* = 0.25), and parathyroidectomy (*RR* = 0.36, 95% *CI* = 0.09 to 1.35, *p* = 0.13) between the Cinacalcet and non-Cinacalcet users. Moreover, Cinacalcet was associated with an increased risk of nausea (*RR* = 2.29, 95% *CI* = 1.73 to 3.05, *p* = 0.001), hypocalcemia (*RR* = 4.05, 95% *CI* = 2.33 to 7.04, *p* = 0.001), and vomiting (*RR* = 1.90, 95% *CI* = 1.70 to 2.11, *p* = 0.001).

**Discussion:**

The number of publications indexed to Cinacalcet and SHPT has increased rapidly over the past 22 years. Literature distribution, research topics, and emerging trends in publications on Cinacalcet and SHPT were analyzed using a machine learning-based bibliometric review. The findings of this meta-analysis provide valuable insights into the efficacy and safety of cinacalcet for the treatment of SHPT, which will be of interest to both clinical and researchers.

## 1 Introduction

Secondary hyperparathyroidism (SHPT) is a medical condition in which a systemic condition outside the parathyroid glands causes all parathyroid glands to become enlarged and hyperactive (1). The most common cause of SHPT is chronic kidney disease (CKD; i.e., a kidney disorder in which a gradual loss of kidney function occurs over a period of months to years; 2). With CKD progression, the kidneys can no longer maintain a calcium and phosphate balance; such changes signal the parathyroid glands to produce excessive amounts of parathyroid hormone (PTH) and grow larger, causing SHPT (3). SHPT is significantly associated with cardiovascular mortality and all-cause mortality (4). Currently, the main treatments for managing SHPT include parathyroidectomy, phosphate binders (i.e., medications used to reduce the absorption of dietary phosphate; 5), vitamin D supplements, and calcimimetics (i.e., drugs that mimic the action of calcium on tissues; 6). Parathyroidectomy, the conventional therapy, is generally a safe procedure; however, as surgery does not treat the disease that causes SHPT, there is a high chance of recurrence; therefore, surgery is not the best option for treating SHPT. If symptoms persist after nonsurgical treatment, a parathyroidectomy may be advised (7). Compared to other treatments, calcimimetics are new but since they play an irreplaceable role in reducing the levels of PTH and serum calcium, calcimimetics are the most widely used treatment strategy for SHPT (8). The *Kidney Disease: Improving Global Outcomes* (KDIGO) guidelines have identified calcimimetics as the first-line therapy for SHPT (9).

Cinacalcet was the first calcimimetic drug to be approved by the United States (U.S.) Food and Drug Administration (10). Cinacalcet is safe and effective in clinical trials, demonstrating superior efficacy in improving bone histology and vascular calcification (11, 12). The advent of cinacalcet has effectively reduced the need for parathyroidectomy (13, 14). Although side effects (e.g., hypocalcemia, nausea, and vomiting) and severe adverse events (e.g., mortality and cardiovascular events) have been reported, the FDA has recently approved more calcimimetics (e.g., Etelcalcetide and Evocalcet) for the treatment of SHPT (15), cinacalcet remains the most prescribed calcimimetic drug (16–18).

Systematic reviews are used to synthesize and criticallyevaluate research findings on a specific topic and are particularly important in the field of medicine to provide evidence-based information that can inform clinical decision-making. Bibliometric analysis, a tool used in systematic reviews, adopts mathematical and statistical methods to demonstrate knowledge structures and dynamic evolution of a specific research area (19). A significant amount of research has been conducted using bibliometric methods (e.g., 20–22). Another classical tool used in systematic reviews is meta-analysis, which combines data from multiple studies to obtain a more precise estimate of treatment effects. By synthesizing the results of multiple studies, a meta-analysis can provide a comprehensive and unbiased evaluation of treatment effectiveness.

Despite the increasing number of studies on Cinacalcet and SHPT, a comprehensive analysis of the existing literature using machine-learning techniques is lacking. Therefore, we conducted a review that included both bibliometric and meta-analyses to comprehensively analyze the literature on Cinacalcet and SHPT. The findings provide comprehensive information regarding the development, hotspots, and future directions within the research fields of Cinacalcet and SHPT. We believe that the insights gained from this study will be of interest to clinicians and researchers.

## 2 Methods

### 2.1 Source of data and searching strategy

Until November 2022, the Web of Science Core Collection database (WoSCC) was searched using the following searching strategy: TS=(‘secondary hyperparathyroidism’ or ‘SHPT’) AND (‘Cinacalcet’ or ‘Mimpara’ or ‘Sensipar’ or ‘AMG 074’ or ‘AMG 073’ or ‘KRN 1493’ or ‘Naphthalene’) AND publishing year= (2000-2022). The raw data were retrieved from the Web of Science Core Collection (WoSCC). The rationale for choosing the WoSCC is that it is a cross-disciplinary data source that can provide more comprehensive information.

### 2.2 Procedure of bibliometric analysis

**Figure 1:**
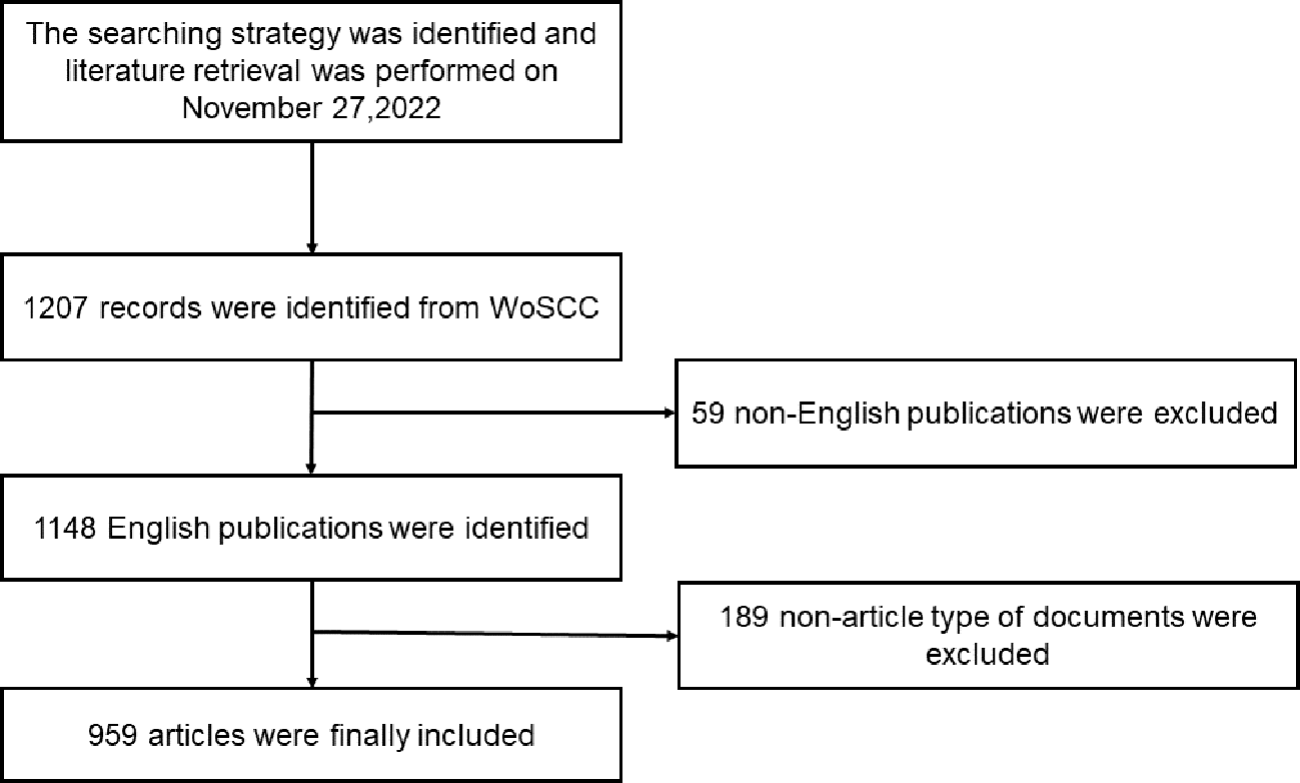
Flowchart of literature screening. WoSCC, Web of Science Core Collection.

The inclusion criteria were as follows: (1) language: English; (2) period: 2000–2022; (3) literature type: articles and reviews. All included studies were exported from WoSCC. Each document record included the article title, keywords, author information, publication date, national sources, and other information. **Figure 1** illustrates the literature screening process used in this study.

The “Bibliometrix” package in the R platform (Version 4.1.2; 23), VOSviewer (Version 1.6.18; 24), and CiteSpace (Version 5.8R3; 25) were used for statistical and visual analysis of literature. Specifically, Bibliometrix was primarily used to analyze documents, count the number of publications and citations, and display cooperation among countries. CiteSpace was used to plot keyword timelines based on the frequency and time of keyword occurrence. VOSviewer was used for network analyses (e.g., collaborative analysis among authors).

We used the Python platform for the Latent Dirichlet Allocation (LDA) theme modeling (26). LDA determines the topic distribution based on the frequency with which vocabulary appears in documents (27). This method has been used in many research areas to identify research topics and trends in publications (e.g., 28, 29). The number of LDA topics was determined based on consistency. The number of topics with a consistency closer to 0.7 in the LDA topic model was considered the ideal (30). The LDA theme model can produce a frequency distribution of topics and theme words. Two of our researchers named each topic based on the distribution of theme words after discussion and analysis.

### 2.3 Procedures of meta-analysis

To be included in the meta-analysis, all articles had to meet the following criteria:1) they must be designed as randomized controlled trials (RCTs); 2) the interventions studied must include both cinacalcet and a control group, and 3) the articles must report clinical outcomes, including serum PTH, calcium, phosphate, and hypocalcemia.

Data extraction was performed independently by two researchers and any discrepancies were resolved through discussion with a third researcher. The recorded data included the author, year, country, intervention, number of participants, age, dosage strategy, the daily dose (minimum and maximum in mg), CKD stage, study duration, follow-up duration (in months), and clinical outcomes such as serum PTH (pg/ml), calcium (Ca; mg/dl), phosphate (P; mg/dl), calcium phosphate products, all-cause mortality, nausea, vomiting, cardiovascular mortality, hypocalcemia, and parathyroidectomy.

The quality of the RCTs was evaluated using the Cochrane Collaboration tool for assessing the risk of bias in randomized trials (31), which assessed the following aspects: random sequence generation, allocation concealment, blinding of patients and study personnel, blinding of outcome assessment, incompleteness of outcome data, selective reporting of outcomes, and other biases.

A meta-analysis was conducted using Review Manager software (version 5.0; The Cochrane Collaboration, Oxford, UK) and STATA software (version 11; Stata Corporation, College Station, TX, USA). For continuous variables (i.e., PTH, calcium, phosphate, and calcium phosphate products), we calculated the mean difference/standardized mean difference (*MD/SMD*) and 95% confidence intervals (95% *CI*). Nominal variables (all-cause mortality, nausea, vomiting, cardiovascular mortality, hypocalcemia, and parathyroidectomy) were analyzed using risk ratios (*RR*) and 95% CI. Heterogeneity was assessed using the *I^2^* statistic, with a random-effects model for *I^2^* greater than 50% and a fixed-effects model for *I^2^* less than 50%. Egger’s test was used to examine publication bias, with statistical significance set at p < 0.05.

## 3 Results

### 3.1 Annual productions

**Figure 2:**
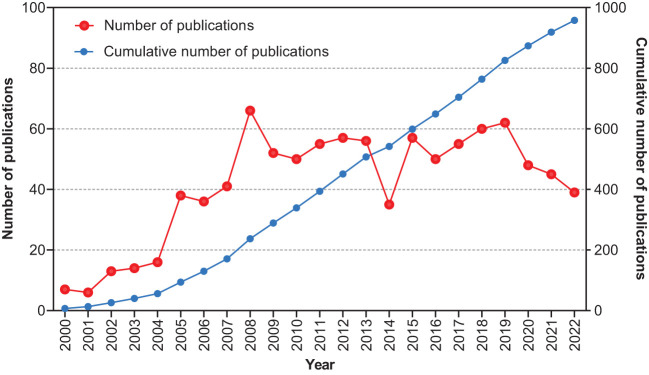
Number of publications and cumulative number of publications.

A total of 959 articles, published between 2000 and 2022, were retrieved. The annual publication rate has shown a growing trend over the past 22 years (**Figure 2**). Only 56 articles were published prior to 2005. A sharp increase was observed in 2005 when the annual number of articles increased to 38. In 2008, this number peaked at 66. The number of outputs remained steady at approximately 50 articles per year.

### 3.2 Countries

Researchers from 54 countries have contributed to this research area. The top ten productive countries were the U.S. (*n* = 267), Japan (*n* = 152), China (*n* = 65), Italy (*n* = 63), Spain (*n* = 63), France (*n* =44), Germany (*n* = 39), the United Kingdom (U.K., *n* = 28), Canada (*n* = 18), and Poland (*n* = 18; **Table 1**).

**Table 1:** The top-ten most productive countries over the period of 2000-2022.

Country	NP	SCP	MCP	MCP Ratio
U.S.	267	209	58	0.217
Japan	152	140	12	0.079
China	65	58	7	0.108
Italy	63	48	15	0.238
Spain	63	45	18	0.286
France	44	30	14	0.318
Germany	39	26	13	0.333
U.K.	28	19	9	0.321
Canada	18	6	12	0.667
Poland	18	17	1	0.056

NP, Number of publications; SCP, Number of single country publication; MCP, Number of multiple countries publication; MCP ratio, MCP as a proportion of total publications.

**Figure 3:**
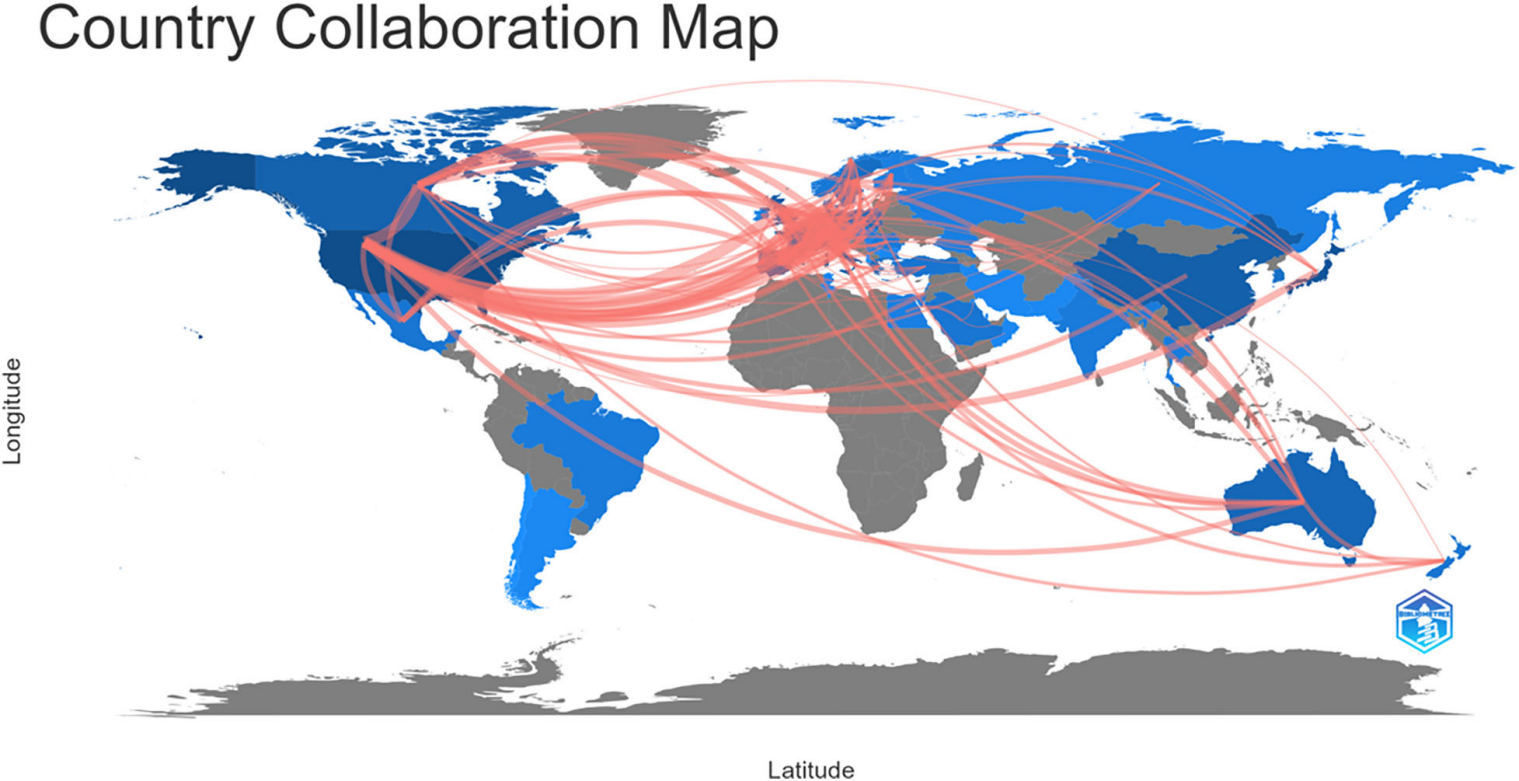
International collaboration map generated by Bibliometrix.

An international collaboration map was generated using the Bibliometrix R package (**Figure 3**; the line thickness indicates the frequency of collaboration among countries). As shown in the Figure, as the most productive country, the U.S. had already built the most international collaborations worldwide, while Japan (i.e., the second most productive country) had only collaborated with a few North American and European countries in this research field.

### 3.3 Institutions

**Figure 4:**
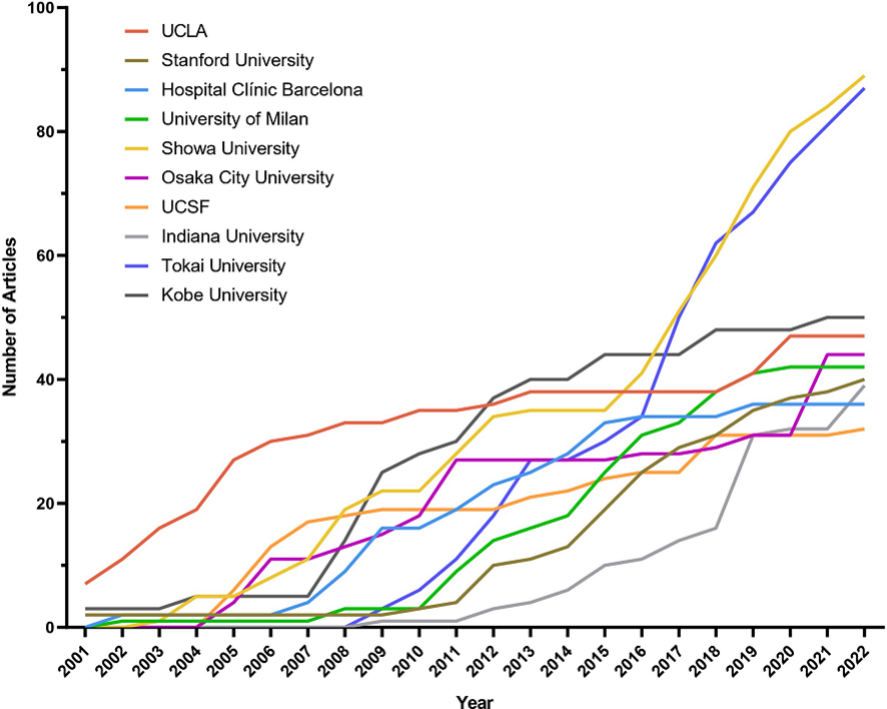
Productivity of the top-ten institutions over the period of 2000-2022. UCLA, University of California Los Angeles; UCSF, University of California San Francisco.

A total of 1416 institutions were involved in Cinacalcet and SHPT research. The top-ten institutions were composed of four Japanese institutions, and four American institutions, while Italy and Spain each had one including Showa University (Japan, *n* = 89), Tokai University (Japan, *n* = 87), Kobe University (Japan, *n* = 50), University of California Los Angeles (UCLA, U.S., *n* = 47), Osaka City University (Japan, *n* = 44), University of Milan (Italy, *n* = 42), Stanford University (U.S., *n* = 40), Indiana University (U.S., *n* = 39), Hospital Clinic Barcelona (Spain, *n* = 36), and University of California San Francisco (U.S., *n* = 32). **Figure 4** shows the institutional productivity over time. UCLA and Kobe University were the two of the earliest to publish studies in this area. Later, Showa University and Tokai University began to pay attention to this field and soon emerged as the most productive institutions.

### 3.4 Authors

**Figure 5:**
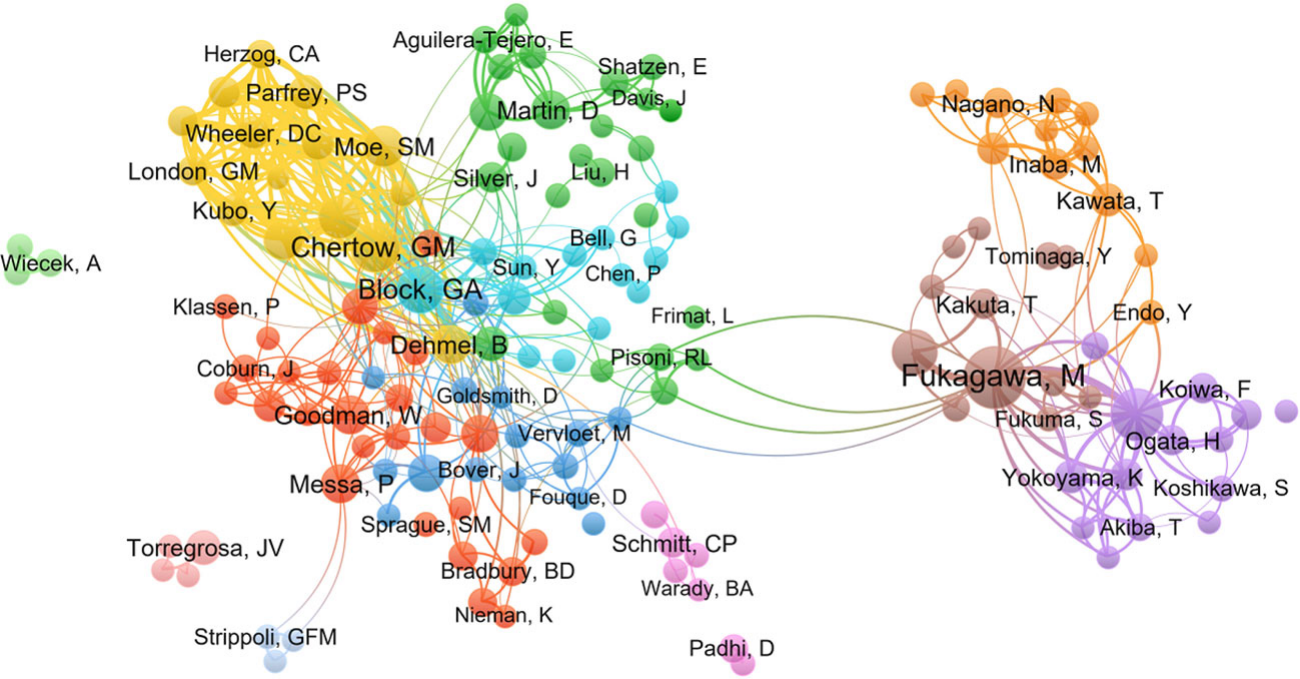
Co-authorship cluster map generated by VOSviewer.

A total of 3573 authors were involved in Cinacalcet and SHPT research over the years. The ten most productive authors are listed in **Table 2**. Dr. William Goodman from the University of California, Los Angeles (U.S.), had the highest h-index (i.e., An author-level citation metric that measures both the productivity and citation impact of publications; 32) and most citations followed by Dr. Misato Fukagawa (Tokai University, Japan) and Dr. Geoffrey Block (Denver Nephrology, U.S.), with h-indexes of 19 and 18, respectively. **Figure 5** shows the collaborations among authors, which can be roughly classified into nine research groups.

**Table 2:** The top-ten most productive authors over the period of 2000-2022.

Author	H_Index	TC	NP	PY_Start
Fukagawa M.	19	1225	50	2008
Chertow G.M.	18	2796	33	2005
Goodman W.G.	21	2899	32	2000
Akizawa T.	12	525	31	2003
Block G.A.	18	2809	25	2003
Komaba H.	14	576	25	2008
Moe S.M.	13	2713	22	2003
Floege J.	13	1695	17	2010
Messa P.	10	549	17	2006
Martin K.J.	13	1697	15	2003

NP, number of publications; TC, Total citations; PY_Start, The year of their first publication in this field.

### 3.5 Journals

In total, 940 articles were published in 320 journals. **Table 3** displays the top ten journals that published the most articles including *Nephrology Dialysis Transplantation* (*n* = 63), *Kidney International* (*n* = 37), *Therapeutic Apheresis and Dialysis* (*n* = 35), *Clinical Journal of the American Society of Nephrology* (*n* = 29), *Clinical Nephrology* (*n* = 25), *BMC Nephrology* (*n* = 20), *American Journal of Kidney Disease*s (*n* = 18), *Journal of the American Society of Nephrology* (*n* = 17), *Pediatric Nephrology* (*n* = 16), and *Plos One* (*n* = 16). *Nephrology Dialysis Transplantation* seems to be the most influential journal in the field with an h-index of 32 and 3098 total citations.

**Table 3:** The top-ten journals published most articles regarding Cinacalcet and secondary hyperparathyroidism research.

Journal	H_Index	TC	NP	PY_Start
Nephrology Dialysis Transplantation	32	3098	63	2002
Kidney International	24	2584	37	2000
Therapeutic Apheresis and Dialysis	13	380	35	2005
Clinical Journal of The American Society of Nephrology	23	1839	29	2006
Clinical Nephrology	12	376	25	2005
BMC Nephrology	9	214	20	2012
American Journal of Kidney Diseases	12	607	18	2004
Journal of the American Society of Nephrology	15	1860	17	2000
Transplantation Proceedings	10	207	16	2006
Pediatric Nephrology	9	226	16	2003

TC, Total citations; NP, number of publications; PY_Start, The year of their first publication in this field.

**Figure 6:**
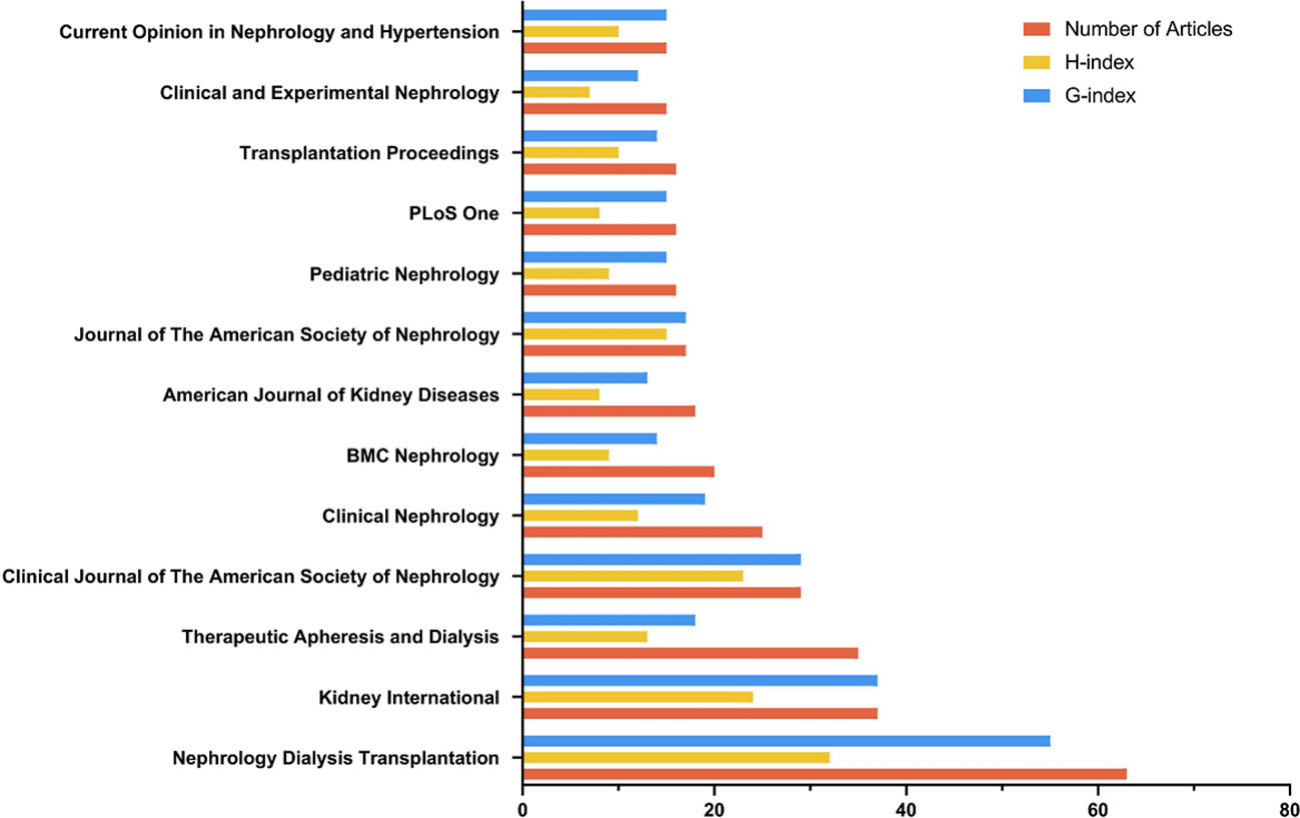
The core journals identified by Bradford’s law. (Bradford’s Law describes the logarithmic distribution of articles across a limited number of core journals in a subject area).

According to Bradford’s law analysis, 13 journals were identified as core journals (**Figure 6**) including *Nephrology Dialysis Transplantation*, *Kidney International*, *Therapeutic Apheresis and Dialysis*, *Clinical Journal of the American Society of Nephrology*, *Clinical Nephrology*, *BMC Nephrology*, *American Journal of Kidney Disease*s, *Journal of the American Society of Nephrology*, *Pediatric Nephrology*, *Plos One*, *Transplanation Proceedings*, *Clinical and Experimental Nephrology*, and *Current Opinion in Nephrology and Hypertension*.

### 3.6 Articles

We used the R bibliometrix package to identify the top ten most-cited articles (**Table 4**). The most cited article was KDIGO 2017 Clinical Practice Guideline Update for the Diagnosis, Evaluation, Prevention, and Treatment of CKD-MBD, an updated guideline for the diagnosis and treatment of CKD-MBD. Of the ten articles, nine were RCTs on the effect of cinacalcet in the treatment of SHPT; the other was a guideline.

**Table 4:** The top-ten most cited articles about Cinacalcet and secondary hyperparathyroidism over the period of 2000-2022.

Article	DOI	Normalized TC
KDIGO 2017 clinical practice guideline update for the diagnosis, evaluation, prevention, and treatment of CKD-MBD	10.1016/j.kisu.2017.04.001	28.26
Cinacalcet for secondary hyperparathyroidism in patients receiving hemodialysis	10.1056/NEJMoa031633	11.11
Effect of Cinacalcet on cardiovascular disease in patients undergoing dialysis	10.1056/NEJMoa1205624	18.80
The ADVANCE study: A randomized study to evaluate the effects of Cinacalcet plus low-dose vitamin D on vascular calcification in patients on hemodialysis	10.1093/ndt/gfq725	16.96
Effects of the calcimimetic Cinacalcet HCl on cardiovascular disease, fracture, and health-related quality of life in secondary hyperparathyroidism	10.1111/j.1523-1755.2005.00596.x	4.64
Cinacalcet HCl, an oral calcimimetic agent for the treatment of secondary hyperparathyroidism in hemodialysis and peritoneal dialysis: A randomized, double-blind, multicenter study	10.1681/ASN.2004060512	4.07
Achieving NKF-K/DOQI bone metabolism and disease treatment goals with Cinacalcet HCl	10.1111/j.1523-1755.2005.67139.x	3.05
The Calcimimetic agent AMG 073 lowers plasma parathyroid hormone levels in hemodialysis patients with secondary hyperparathyroidism	10.1681/ASN.V1341017	4.62
Cinacalcet, fibroblast growth factor-23, and cardiovascular disease in hemodialysis: The EVOLVE Trial	10.1161/CIRCULATIONAHA.114.013876	7.49
The calcium-sensing receptor in normal physiology and pathophysiology: a review	10.1080/10408360590886606	2.62

TC, Total citations; KDIGO, The kidney disease: improving global outcomes; CKD-MBD, Chronic kidney disease - mineral and bone disorder; ADVANCE, Assessing donor variability and new concepts in eligibility; HCI, Cinacalcet hydrochloride; EVOLVE, Evaluation of Cinacalcet HCl therapy to lower cardiovascular events.

### 3.7 Topic modeling

**Figure 7:**
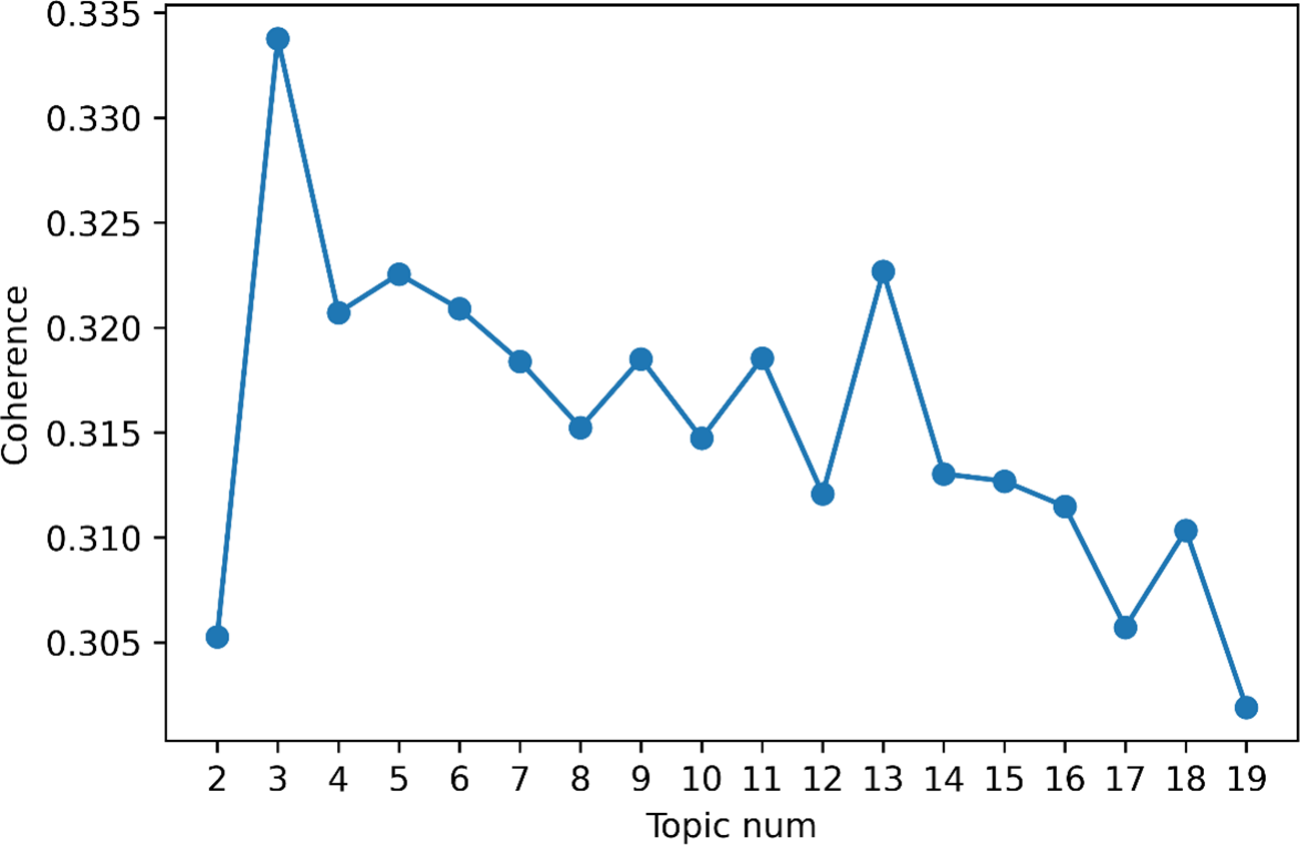
Number of topics-coherence score graph.

**Figure 8:**
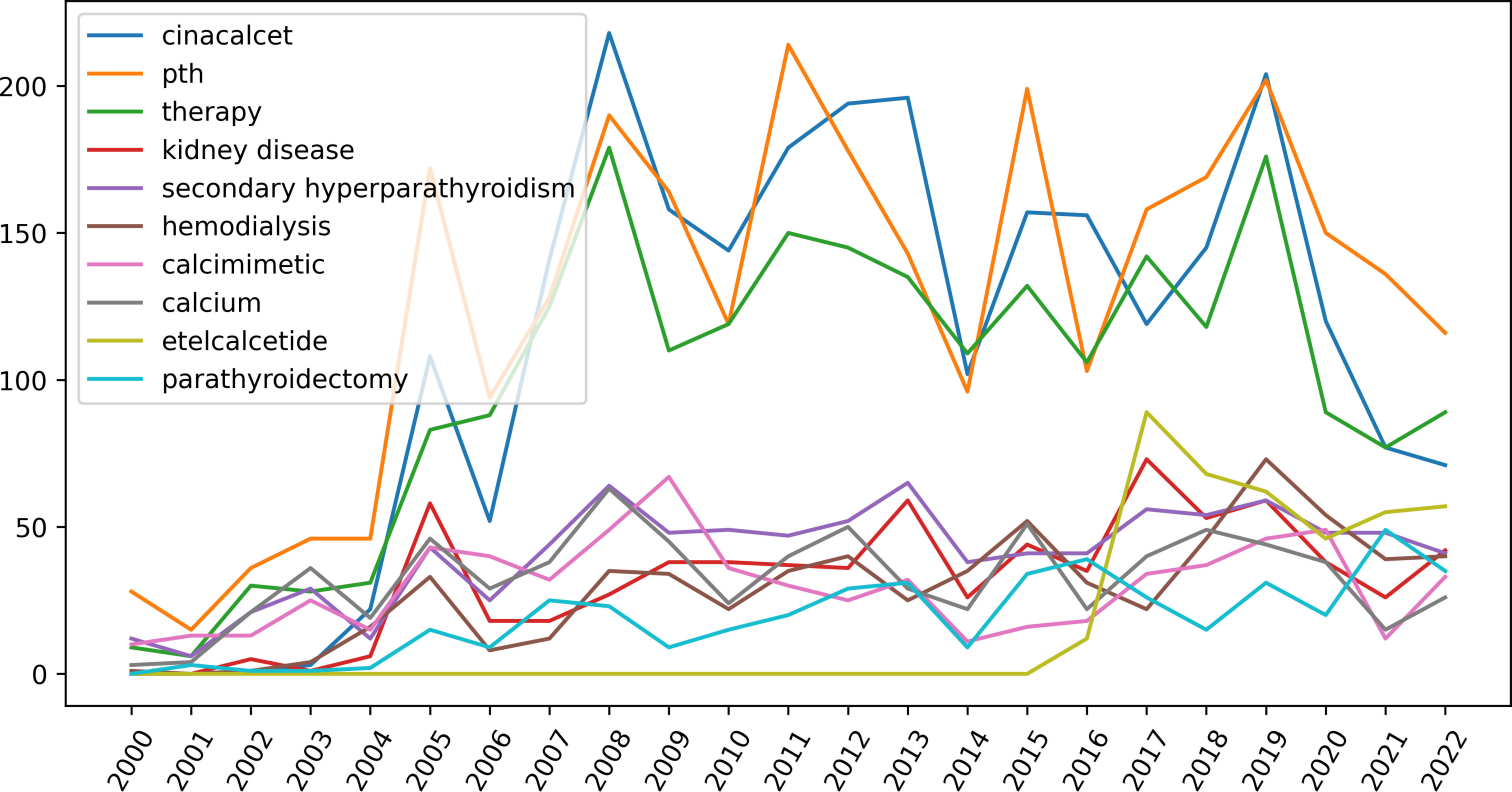
The development of the top-ten keywords generated by Latent Dirichlet Allocation (LDA) analysis. (LDA is a topic modeling method being used to determine hidden themes from large texts).

Topic modeling can classify themes and discover hidden themes (33). We determined that the optimal number of topics for this study was three (**Figure 7**). By applying LDA-based topic analysis, we identified the three most popular topics within this research field (**Table 5**) including Topic 1, the comparative effects of various treatments: cinacalcet, telcalcetide, calcimimetic, parathyroidectomy, Vitamin D, Evocalcet; Topic 2, safety and efficacy: PTH level, serum calcium, serum phosphate, vascular calcification, cardiovascular events, and mortality, and Topic 3, fibroblast growth factor-23 (FGF-23), paricalcitol, phosphate, and calcitriol. A time distribution analysis was then performed to detect the development of the top-ten keywords (**Figure 8**).

**Table 5:** Research topics generated by Latent Dirichlet Allocation analysis.

Topic		Keywords
1	Comparative effectiveness	Cinacalcet, Etelcalcetide, Evocalcet, Parathyroidectomy, Vitamin D, Therapy, Hemodialysis Patients, Kidney Disease, Secondary Hyperparathyroidism, Clinical Trial, Calcimimetic, PTH, Serum Calcium, Cardiovascular Events, Vascular Calcification, Complication, Nausea, Vomit, Hypocalcemia, Mortality
2	Safety and efficacy	Cinacalcet, Secondary Hyperparathyroidism, Therapy, Calcimimetic, Hemodialysis, Calcium, Phosphate, PTH, Parathyroidectomy, Management, Kidney Disease, Complication, Hypocalcemia, Mortality, Calcification, Hyperplasia, Parathyroid, Alkaline Phosphatase, Calcium-Sensing Receptor, Trial
3	FGF-23	PTH, Kidney Disease, Therapy, Secondary Hyperparathyroidism, FGF-23, Cinacalcet, Hemodialysis, Vitamin D, Calcium, Phosphate, Mortality, Bone Disease, Marker, Management, CKD-MBD, Kidney, Skeletal, Complication, Cardiovascular Events, End-Stage Renal Disease

FGF-23, fibroblast growth factor-23; PTH, parathyroid hormone; CKD-MBD, Chronic kidney disease - mineral and bone disorder.

### 3.8 Citation burst

**Figure 9:**
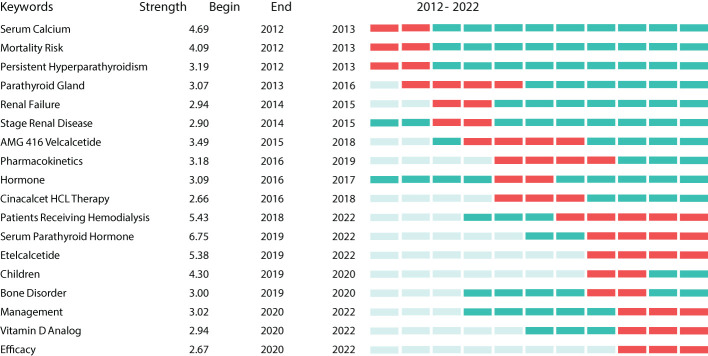
Strongest citation burst generated by CiteSpace.

Burst keywords can be regarded as indicators of emerging trends (25). Burst detection analysis was conducted using CiteSpace software. **Figure 9** shows the 18 keywords with the strongest citation bursts between 2012 and 2022 (red indicates the time at which a citation burst was identified). The most recent burst keywords were patients receiving hemodialysis, serum PTH, etelcalcetide, children, bone disorder, management, Vitamin D analog, and efficacy.

### 3.9 Meta-analysis

We conducted a meta-analysis of 24 RCTs with 9130 participants. The characteristics of the retrieved studies are presented in **Table 6**. A summary of the selection bias is presented in **Supplementary 1**, in which all included articles demonstrated high quality and low risk of bias. Egger’s test was used to assess the possibility of publication bias. Funnel plot analysis did not reveal any significant publication bias (**Supplementary 2**).

**Table 6:** Characteristics of meta-analysis included studies.

Author	Year	Country	Stage of CKD	Arm1	Arm2	Age1	Age2	Duration of the trial	Outcome	Follow up
Akiba	2008	Japan	HD	Cinacalcet, 12.5、25、50 mg/d,N=90	Placebo, N = 30	N	51.8 ± 7.5	3 weeks	Serum PTH, Ca,P,calcium-phosphate product,nausea, vomiting, hypocalcemia and Cardiac disorders	2 weeks
Goodman	2000	USA	HD	R-568, 100 mg/d,N=16	Placebo, N = 5	48.6 ± 12.4	54.7 ± 16.8	15 days	Serum PTH, nausea and hypocalcemia	0.5 months
Goodman	2002	USA	HD	AMG 073, 10–50 mg/d, N=23	Placebo, N = 7	N	N	8 days	Serum PTH, Ca and P	0.25 months
Lindberg	2003	USA	HD	AMG 073, 10–50 mg/d, N=38	Placebo, N = 39	52.7 ± 16.4	48.8 ± 15.6	4.5 months	All-cause mortality,pth, Ca, P,nausea and vomiting	4.5 months
Quarles	2003	USA	HD	AMG 073, 25–100 mg/d,N=36	Placebo, N = 35	49.6 ± 8.5	47.9 ± 14.2	18 weeks	Serum PTH, Ca,P,calcium- phosphate product	4.5 months
Block	2004	USA	HD	Cinacalcet, 30–180 mg/d,N = 371	Placebo, N = 370	54 ± 14	55 ± 15	26 weeks	Serum PTH, Ca,P,calcium- phosphate product,nausea, vomiting, hypocalcemia, hypotension and all-cause mortality	6.5 months
Charytan	2005	USA	CKD,not receiving dialysis	Cinacalcet, 30–180 mg/d, N = 27	Placebo, N = 27	60.6 ± 15.6	61.9 ± 15.1	18 weeks	Serum PTH, nausea,Cardiovascular mortality and all-cause mortality	4.5 months
Lindberg	2005	USA	HD PD	Cinacalcet, 30–180 mg/d,N = 371	Placebo, N = 101	51.8 ± 14	53.5 ± 13.9	26 weeks	Serum PTH, calcium, phosphate, calcium phosphate product, all-cause mortality, nausea, and vomiting.	6.5 months
Fishbane	2008	USA	HD	Cinacalcet,30–180 mg/d plus paricalcitol 2 g or doxercalciferol 1 g,N =87	Paricalcitol 2 g or doxercalciferol 1 g,N=86	57.7 ± 14.9	59 ± 12.4	27 weeks	All-cause mortality, nausea, vomiting, hypercalcemia, and hypocalcemia.	6.75 months
Fukagawa	2008	Japan	HD	Cinacalcet, 30–180 mg/d;N = 72	Placebo, N = 71	54.7 ± 11	55.7 ± 11.7	14 weeks	Serum calcium, phosphate, calcium phosphate product, nausea, vomiting, and hypocalcemia	3.5 months
Messa	2008	Italy	HD	Cinacalcet, 30–180 mg/d,N = 368	Conventional Care,N = 184	58.5 ± 14.5	58.3 ± 14.5	23 weeks	Serum PTH, calcium, phosphate, calcium phosphate product, all-cause mortality, cardiovascular mortality, nausea, vomiting, and hypocalcemia.	0.25 months
Chonchol	2009	USA	CKD,not receiving dialysis	Cinacalcet, 30–180 mg/d, N = 302	Placebo,N = 102	64.7 ± 13.3	66.2 ± 12.2	32 weeks	Serum calcium, phosphate, calcium phosphate product, all-cause mortality, cardiovascular mortality, nausea, vomiting, and hypocalcemia.	8 months
EI-Shafey	2011	Egypt	HD	Cinacalcet, 30–180 mg/d,N = 55	conventional therapy,(intravenous alfacalcidol thrice weekly at the end of their dialysis session and phosphate binders), N = 27	51.5 ± 12.7	51.8 ± 15	36 weeks	Serum PTH, calcium, phosphate, calcium phosphate product, all-cause mortality, nausea, vomiting, and hypocalcemia.	9 months
Raggi	2011	USA	HD	Cinacalcet, 30–180 mg/d plus low-dose vitamin D,N = 180	Same dose of vitamin D prescribed N = 180	61.2 ± 12.6	61.8 ± 12.8	52 weeks	All-cause mortality and hypocalcemia.	12 months
Chertow	2012	USA	HD	Cinacalcet, 30–180 mg/d,N = 1948	Placebo, N = 1935	55.0 (35–74)	54.0 (35–73)	20 weeks	All-cause mortality, cardiovascular mortality, nausea, vomiting and hypocalcemia.	64 months
Ketteler - intra venous stratum	2012	Germany	HD	Cinacalcet (dose unclear) plus low-dose vitamin D,N = 64	Paricalcitol 0.07 μg/kg IV or iPTH/60 PON = 62	59.9 ± 12	61.2 ± 12.7	28 weeks	All-cause mortality, cardiovascular mortality, nausea, vomiting, hypercalcemia, and hypocalcemia.	7 months
Ketteler- 2-oral stratum	2012	Germany	HD	Cinacalcet (dose unclear) plus low-dose vitamin D,N = 70	Paricalcitol 0.07 μg/kg IV or iPTH/60 PON = 72	65.1 ± 12.5	65.7 ± 13.5	28 weeks	All-cause mortality, cardiovascular mortality, nausea, vomiting and hypocalcemia.	7 months
Kim	2013	Korea	HD	Cinacalcet, 25–50 mg/d plus low-dose vitamin D,N =33	Same dose of vitamin D prescribed N = 33	48.8 ± 11.5	47.2 ± 8.4	20 weeks	Serum PTH, nausea and hypocalcemia.	4 months
Urena-Torres	2013	USA	HD	Cinacalcet, 25–50 mg/d plus low-dose vitamin D,N =154	Same dose of vitamin D prescribed N = 155	57.9 ± 13.6	57.0 ± 14.6	52 weeks	All-cause mortality, nausea, vomiting and hypocalcemia.	12 months
Wetmore	2015	USA	HD	Cinacalcet, 30–180 mg/d,N = 155	vitamin D (dose unclear),N = 157	53 (21–81)	55 (22–86)	52 weeks	Serum PTH, calcium, phosphate, all-cause mortality and hypocalcemia.	24 months
Mei	2016	China	HD	Cinacalcet, 25–100 mg/d,N = 118	Placebo, N = 114	50.02 ± 11.17	50.12 ± 11.34	16 weeks	Serum PTH, calcium, phosphate, calcium phosphate product, nausea, vomiting, and hypocalcemia.	3.4 months
Akizawa	2018	Japan	HD	Cinacalcet, 25 mg/d,N = 30	Placebo, N = 30	58.1 ± 10.2	58.2 ± 10.4	30 weeks	cardiovascular mortality,nausea and hypocalcemia	0.75 months
Fugakawa	2018	Japan	HD	Cinacalcet, 25-100 mg/d,N = 317	Evocalcet, 1-8 mg/d,N = 317	61.2 ± 11	61.5 ± 11.3	30 weeks	nausea, vomiting and hypocalcemia.	7.3 months
Susantitaphong	2019	Thailand	HD	Cinacalcet, 25-100 mg/d,N = 15	Placebo, N = 15	49.5 ± 11.9	49.4 ± 10.2	12 weeks	Serum PTH, calcium and phosphate	2.7 months
Eddington	2021	England	HD	Cinacalcet, 30-180 mg/d,N = 15	Placebo, N = 21	45 ± 16	54 ± 13	12 weeks	Serum PTH, calcium, phosphate, all-cause mortality and hypocalcemia.	12 months

CKD, Chronic kidney disease; HD, hemodialysis.

**Figure 10:**
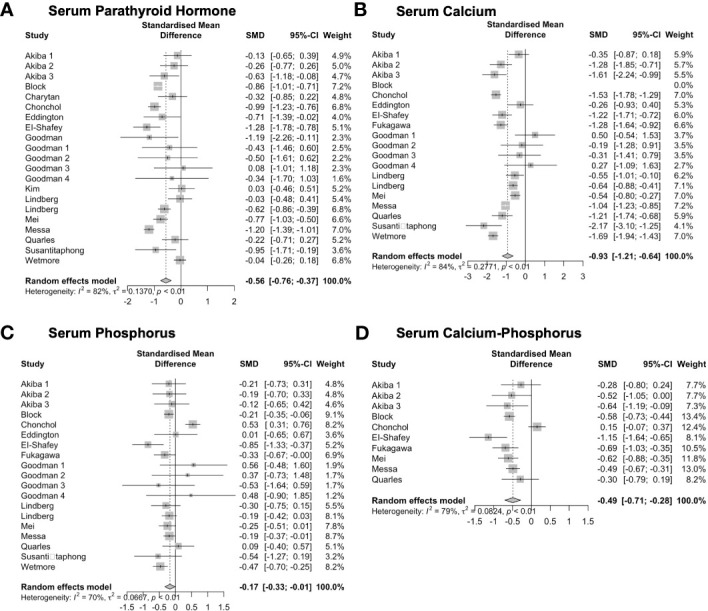
Forest plots demonstrating the effects of Cinacalcet on **(A)** serum parathyroid hormone, **(B)** calcium, **(C)** phosphate, and **(D)** calcium- phosphate.

The effects of Cinacalcet on serum indicators, including PTH, calcium, phosphate, and calcium-phosphate, were evaluated in this study (see **Figure 10**). Twenty-one studies comprising 3280 observations, were included in a pairwise meta-analysis to investigate the effects of Cinacalcet on serum PTH levels. The random-effects model revealed a statistically significant *SMD* of -0.56 (95% *CI* = -0.76, -0.37, *z* = -5.57, *p* = 0.001). Substantial heterogeneity was observed among the studies (*I* ^2 =^ 82.0%, *p* = 0.001). The calcium analysis included 18 studies comprising 3282 observations. The random-effects model revealed a significantly negative *SMD* of -0.93 (95% *CI* = -1.21 to -0.64; *z* = -6.44, *p* = 0.001), indicating a moderate effect size in favor of the intervention. Heterogeneity was high (*I* ^2 =^ 84.5%, *p* < 0.01). Based on the analysis of phosphate, the random effects model found a significant overall effect size of -0.17 (95% *CI* = -0.33 to -0.01, *z* = -2.13, *p* = 0.033). Heterogeneity analysis revealed a moderate-to-high level of heterogeneity among the studies (*I* ^2 =^ 70.0%, *p* < 0.01), indicating a substantial variation in effect sizes across studies. Ten studies were included in the analysis of serum calcium-phosphate product levels, comprising 2388 observations. The random-effects model showed a significant overall effect of the intervention (*SMD* = -0.49, 95% *CI* = -0.71, -0.28, *z* = -4.55, *p* = 0.001), indicating a moderately beneficial effect. However, there was significant heterogeneity among the studies (*I* ^2 =^ 79.1%, *p* = 0.001), indicating that the effect size estimates varied considerably.

**Figure 11:**
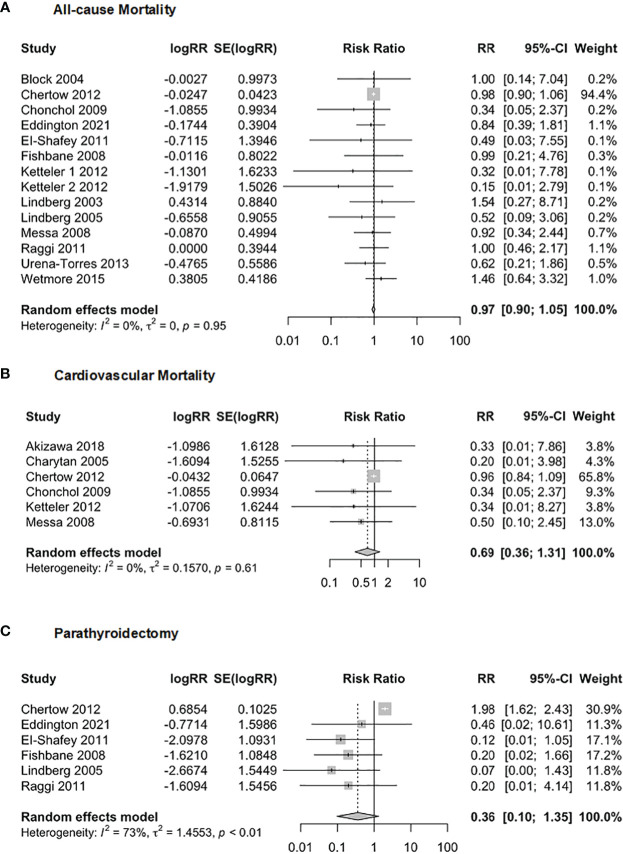
Forest plot demonstrating the association between Cinacalcet and risk for **(A)** allcause mortality, **(B)** cardiovascular mortality, and **(C)** parathyroidectomy.

We investigated the relationship between Cinacalcet and adverse events, including all-cause mortality, cardiovascular mortality, and parathyroidectomy (**Figure 11**). In terms of the relationship between Cinacalcet and all-cause mortality (*n* = 7586), our random effects model yielded a pooled *RR* of 0.97, 95% *CI* = 0.90 to 1.05], *z* = -0.73, *p* = 0.47. There was no significant difference in all-cause mortality observed in individuals taking Cinacalcet. Additionally, there was no evidence of heterogeneity across studies, with *I* ^2 =^ 0.0%, *p* = 0.95. Our analysis of the association between Cinacalcet use and cardiovascular mortality showed no significant differences between groups. The pooled *RR* estimate was 0.69 (95% *CI*:0.36 to 1.31, *p* = 0.25). The test for heterogeneity was not statistically significant (*I* ^2 =^ 0.0%, *p* = 0.612), suggesting a low heterogeneity among the studies. Six studies with a total of 4901 participants were included to investigate the association between cinacalcet and parathyroidectomy. The random effects model showed a pooled *RR* of 0.36, 95% *CI* = 0.09 to 1.35, *z* = -1.51, *p* = 0.13, indicating no significant difference in parathyroidectomy between groups. The test for heterogeneity showed moderate heterogeneity between the studies, with an *I* ^2^ value of 72.6%, *p* < 0.01.

**Figure 12:**
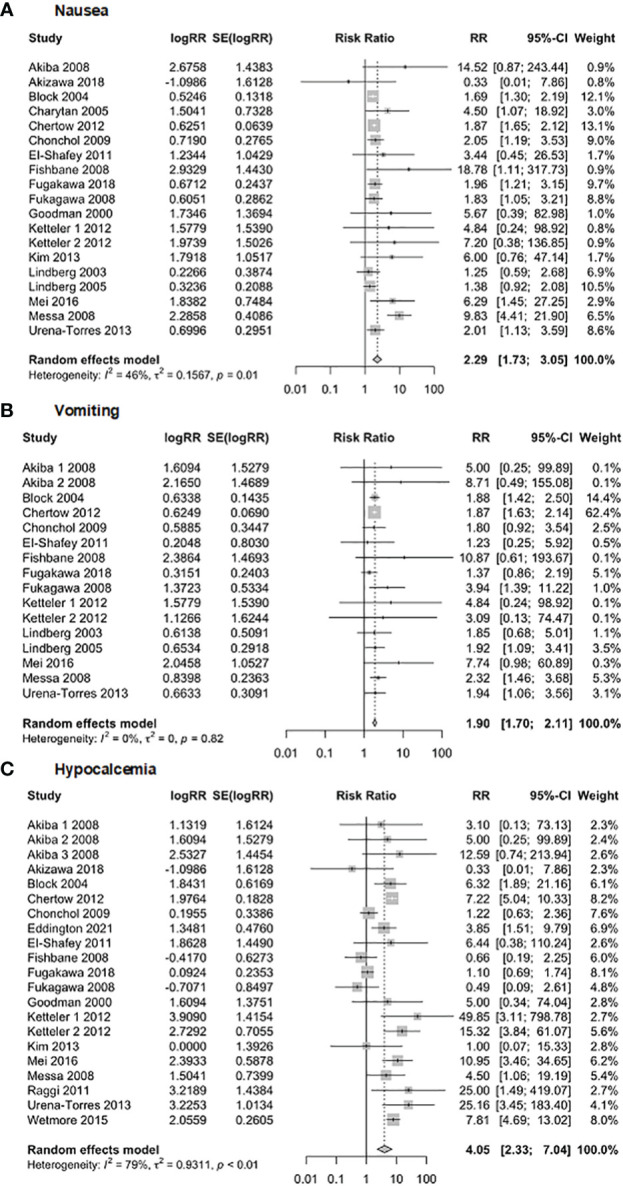
Forest plot demonstrating the safety of Cinacalcet: **(A)** nausea, **(B)** vomiting, and **(C)** hypocalcemia.

The safety of Cinacalcet was evaluated in this meta-analysis (**Figure 12**) by examining its association with nausea, vomiting, and hypocalcemia. The analysis of nausea included 19 studies with 8,127 observations. The results showed a random effects model *RR* of 2.29 (95% *CI* of 1.73 to 3.05, *p* = 0.001), indicating a statistically significant association between Cinacalcet use and nausea. The heterogeneity test revealed significant heterogeneity (*I* ^2 =^ 46.4%, *p* = 0.01). The analysis of vomiting included 16 studies with 7,986 observations, and the random-effects model produced a pooled *RR* of 1.90 (95% *CI* = 1.70, 2.11, *p* = 0.001). The heterogeneity test revealed no significant heterogeneity (*I* ^2 =^ 0%; *p* = 0.82). Finally, the analysis of hypocalcemia included 21 studies with 8,376 observations, and the random effects model produced a pooled *RR* of 4.05 (95% *CI* = 2.33 to 7.04, *p* = 0.001). The heterogeneity test revealed significant heterogeneity (*I* ^2 =^ 79%, *p* < 0.01).

## 4 Discussion

We conducted a systematic review, including bibliometric and meta-analyses, to comprehensively analyze the literature on Cinacalcet and SHPT. This bibliometric review mapped the trends and patterns of Cinacalcet and SHPT publications by applying machine learning methods, further enriching previous reviews. This study captured the growth of these publications and the characteristics of the literature distribution, suggesting the critical role of Cinacalcet in SHPT research. Moreover, we applied advanced topic and keyword modeling methods to thoroughly understand the content of publications and explore the development of research topics and emerging trends. By applying LDA-based topic modeling analysis, three topics were generated including comparative effectiveness, drug safety assessment, and FGF-23.

Topic 1 mainly covered comparisons between Cinacalcet and other treatments. Parathyroidectomy, oral medications (e.g., Evocalcet and Cinacalcet), and intravenous medications (e.g., Etelcacetide and Upasita) are the three major treatment strategies for SHPT. Parathyroidectomy is the conventional treatment for SHPT. Compared to drugs, parathyroidectomy is better at reducing the level of serum calcium (14). However, parathyroidectomy may lead to low calcium levels, and there is also a risk of recurrence; therefore, surgery is typically not the first option (9). The availability of Cinacalcet has reduced the need for parathyroidectomy; however, surgery remains irreplaceable in patients with severe SHPT or who are drug-resistant (14). As the first FDA-approved calcimimetic medication, Cinacalcet is safe and effective. The major limitation of Cinacalcet is its gastrointestinal side effects, with approximately 15–30% of users reporting symptoms of nausea and/or vomiting (13). This is largely due to oral administration; therefore, the newly released calcimimetics, Etelcalcetide and Upasita, are both intravenously administered (34, 35). The comparison of Cinacalcet with other treatments is a research hotspot in this field. Through topic modeling analysis, we also noticed that combined therapies are increasingly being focused on. In recent years, researchers have conducted multiple trials to compare the efficacy of Cinacalcet plus supplements (e.g., Vitamin D and Calcitriol) versus Cinacalcet alone in the treatment of SHPT in recent years (36–38).

Topic 2 was generally safety and efficacy. PTH is a peptide hormone secreted by the parathyroid glands that controls the levels of serum calcium, phosphate, and vitamin D (6). Phosphate and calcium imbalances result in the secretion of excessive amounts of PTH, which is the primary pathological manifestation of SHPT (39). Elevated PTH levels are strongly associated with higher mortality and cardiovascular events in patients with CKD (39). The effectiveness of Cinacalcet in reducing PTH levels, thereby lowering the risk of death and cardiovascular events, has been confirmed by some researchers (11, 40). However, in some studies, Cinacalcet has not demonstrated superior efficacy compared with conventional treatments (41). The role of Cinacalcet in reducing adverse events remains inconclusive and has always been an important focus in this field. Researchers have also suggested that these effects may be moderated by the patient’s age (17). In their study, Cinacalcet effectively decreased the risk of adverse events in older patients but not the younger individuals (17). This finding suggests that more demographic factors should be examined in future studies. Moreover, the European Medicines Agency recently approved the use of Cinacalcet in children aged > 3 years on dialysis (until then, Cinacalcet was used only for adults; 42). Given the requirements of growth and development, many more factors need to be monitored and managed in pediatric SHPT (42). Although no approval for its use has been found in any region other than Europe, according to existing literature, Cinacalcet for pediatric use has attracted the attention of practitioners worldwide. The keyword citation burst results support this view.

Topic 3 was mostly related to FGF-23, which is an emerging hotspot. FGF-23 plays a central role bone-kidney-parathyroid axis and is associated with mortality and cardiovascular events in CKD (43, 44). FGF-23 is a bone-derived hormone that suppresses phosphate reabsorption and Vitamin D hormone synthesis in the kidney, and the resulting Vitamin D deficiency reduces calcium absorption and CaSR-mediated PTH inhibition (45, 46). FGF-23 is therefore regarded as a risk factor of CKD (47). Cinacalcet is effective in controlling PTH levels by inhibiting FGF-23 secretion and thus may play a role in decreasing the incidence of adverse events (48, 49). Moreover, in patients with end-stage renal disease, controlling FGF-23 has recently been found to be more effective in reducing cardiovascular events than controlling PTH (50, 51). FGF-23 is considered a new therapeutic target and is expected to be a promising field of investigation in the future (52).

Based on these findings, we conducted a meta-analysis to examine the clinical efficacy, adverse events, and safety of Cinacalcet. The results of this meta-analysis confirm the effects of Cinacalcet in patients with SHPT. Specifically, Cinacalcet use was associated with significant reductions in serum PTH, calcium, phosphate, and calcium-phosphate levels, indicating its beneficial effects on bone and mineral metabolism. However, significant heterogeneity was observed among the studies, suggesting that the effect of cinacalcet on these indicators may vary depending on patient characteristics, treatment protocols, and other factors. In terms of safety, the analysis found that Cinacalcet is associated with an increased risk of nausea, vomiting, and hypocalcemia. Although these adverse effects are generally mild to moderate in severity, they may limit the clinical utility of Cinacalcet in some patient populations. However, the overall incidence of adverse events associated with Cinacalcet is relatively low, suggesting that the benefits of the drug may outweigh its risks for many patients.

This study has a few limitations. First, only the WoSCC database was searched, which may have led to bias. Detailed and comprehensive knowledge can be obtained if other databases (e.g., Scopus and PubMed) are explored. Second, limited by the length of the journal manuscript, we cannot present all the results of our analyses (e.g., all the countries, authors, keywords, and citations). However, some information may have been missing from our study. Third, the meta-analysis included studies with significant variability in terms of patient populations, treatment protocols, and outcome measures, thus likely limiting the generalizability of these findings. Finally, although quantitative metrics reflect the popularity of scientific research, the results should be interpreted carefully.

## 5 Conclusions

For the first time, this systematic review included both bibliometric and meta-analytical methods that analyzed current publications on Cinacalcet and SHPT through machine learning, which is expected to be helpful for researchers in extracting objective and comprehensive clues from large amounts of data. Researchers from the U.S. and Japan started the earliest and made the greatest contributions to this field. The comparative effects of various treatments, safety and efficacy, and FGF-23 are the three major topics in this research field. Integrated treatment, Cinacalcet use in pediatric CKD, and new therapeutic targets are novel topics that have received increasing attention from researchers and clinicians. Through a meta-analysis, we confirmed that Cinacalcet was effective in reducing serum PTH and calcium levels and improving serum phosphate and calcium- phosphate levels; however, there was no difference in all-cause mortality, cardiovascular mortality, and parathyroidectomy between Cinacalcet and non-Cinacalcet users. Moreover, Cinacalcet is associated with an increased risk of nausea, hypocalcemia, and vomiting. These findings have important implications for the management of patients with SHPT and highlight the potential benefits and risks of cinacalcet.

## Data availability statement

The original contributions presented in the study are included in the article/**Supplementary Material**. Further inquiries can be directed to the corresponding author.

## Author contributions

Two authors, XL and WD, designed this study and collected and processed relevant data. The manuscript was written by XL and HZ. All authors have contributed to the article and approved the submitted version.
