# Supplementary material for: Cinacalcet use in secondary hyperparathyroidism: a machine learning-based systematic review
Source: Front Endocrinol (Lausanne). 2023 Jul 19;14:1146955. doi: 10.3389/fendo.2023.1146955 (PMC10395090; doi:10.3389/fendo.2023.1146955)
Supplement: Supplementary file 1 [file DataSheet_1.pdf]

S1. Quality assessment for the risk of bias

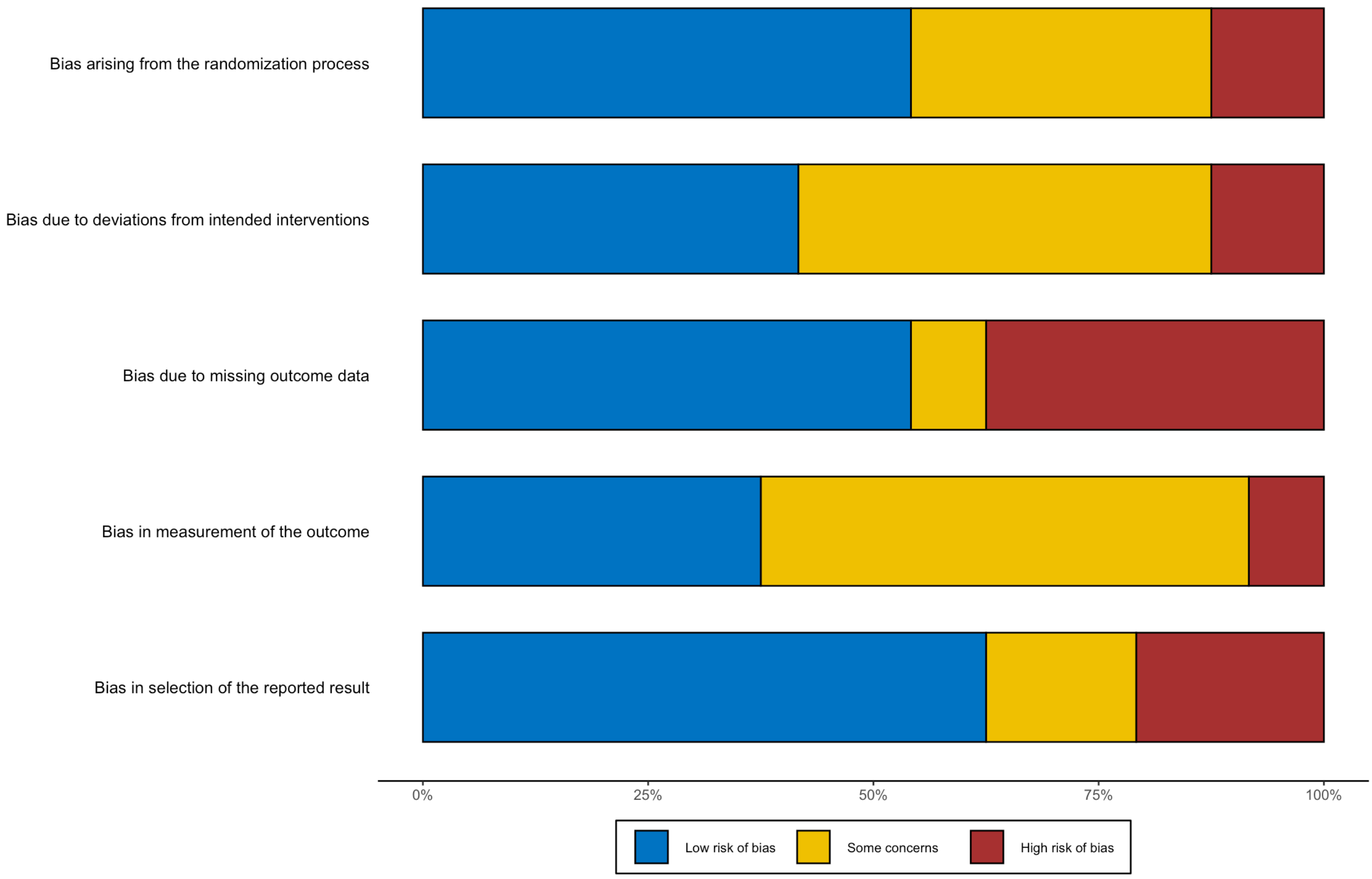

S2. Funnel plots of publication bias

Serum Parathyroid Hormone

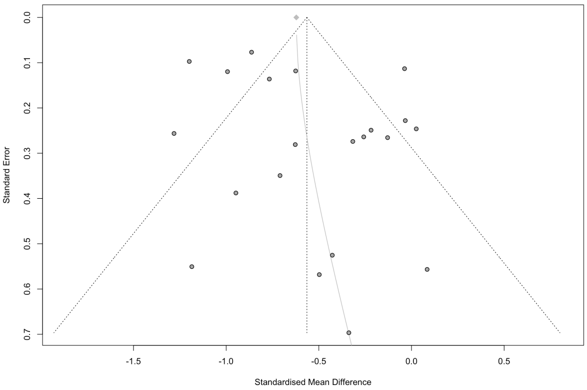

Serum Calcium

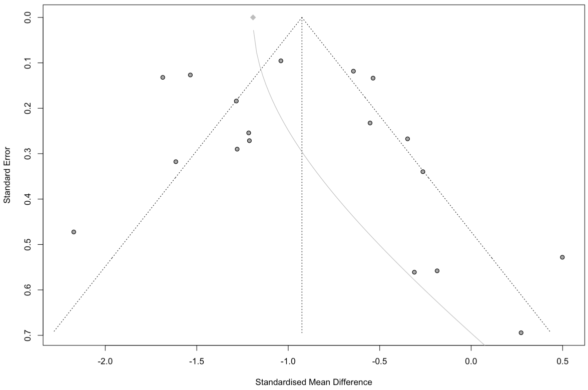

Serum Phosphorus

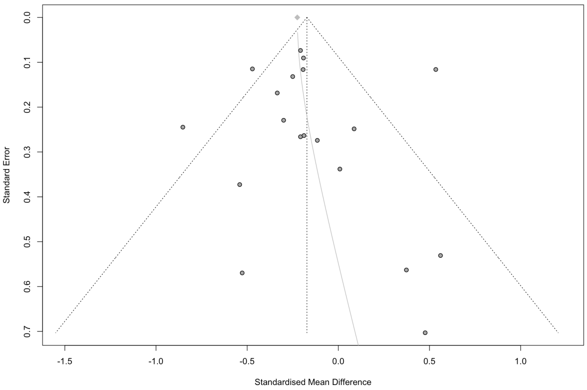

Serum Calcium-Phosphorus

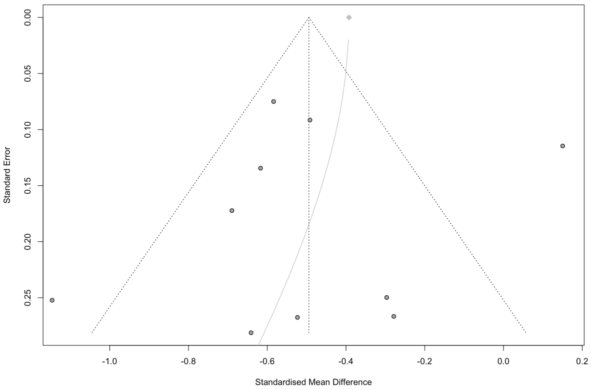

Nausea

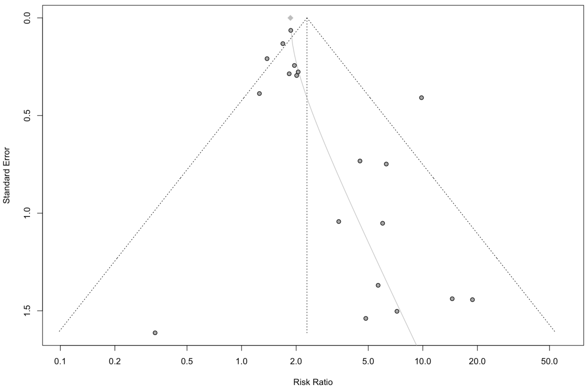

Vomiting

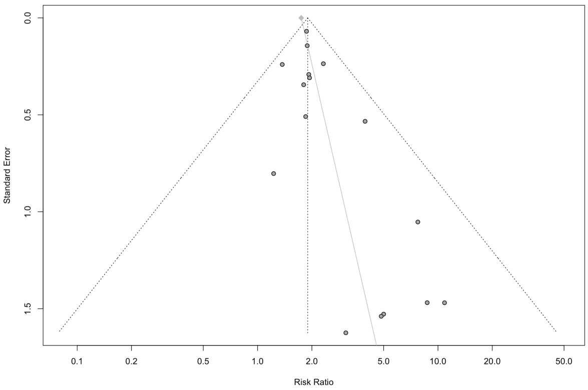

Hypocalcemia

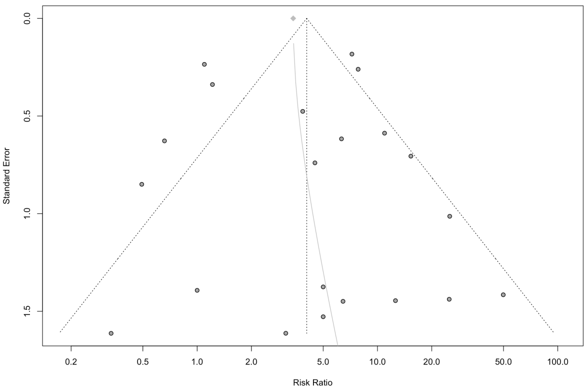

All-cause mortality

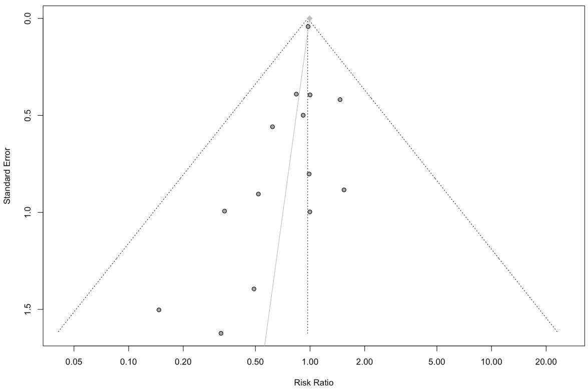

Cardiovascular mortality	Parathyroidectomy
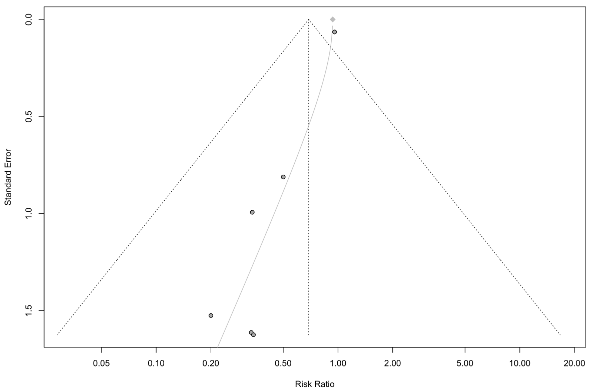	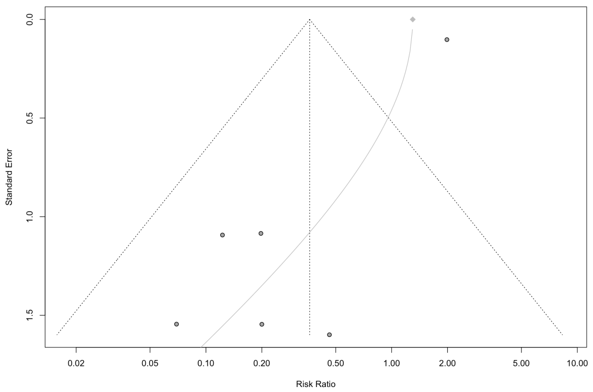
